# Tuberculosis and the General Hospital

**Published:** 1913-07-05

**Authors:** Thomas Oliver


### Tuberculosis and the General Hospital.

By SIR THOMAS OLIVER, M.D., LL.D., D.Sc., F.R.C.P.

(Read at the Oxford Conference in the author's absence by the Rev. G. B. Cronshaw.)

The discovery of the tubercle bacillus by Koch gave not only a fresh impetus to the study of preliminary and other forms of tuberculosis, but it united all the forms together and raised hopes that the malady would be brought within the sphere of prevention and cure. Since it is of all maladies the one disease which claims the greatest number of victims—50,000 annually in this country, or one-ninth of the total death-rate—the problem of tuberculosis remains for medical men one of the most fascinating of our time. In the wards of our general hospitals there are almost always cases of tuberculosis. With our increasing knowledge of the disease and of its infectious nature, we naturally ask ourselves whether it is desirable that such a disease should be treated in general hospitals. It seems something of a contradiction that, while hygienists are teaching the necessity of segregating phthisical patients in their homes or of removing them to sanatoria, hospital physicians should be treating them in their wards. The experience of the Brompton Hospital is quoted to show that, given good conditions and plenty of ventilation, the infectiousness of tubercle is slight. Medical practitioners also tell us that only in a few instances, considering the opportunities for infection, is the disease directly conveyed from a sick husband to a healthy wife, and *vice versa*. Opposed to these there are instances on record of nearly whole families having been wiped out by the introduction into the home of an infected member. The case quoted by Dr. H. G. Sutherland * of the serious consequences which followed the introduction of illness by a sick daughter into the home of a crofter at Tarbert, Morar, cannot be ignored. The father, who was a ghillie, had lived in the same house for twenty-one years. His wife was healthy and was the mother of seven sons and five daughters, their ages ranging from twenty-one to two years. In April 1906 the eldest girl, aged twenty-one, who had been in service, came home suffering from a suppurating finger. Shortly afterwards symptoms of phthisis showed themselves, and she succumbed to tubercular meningitis on May 26. Six months afterwards a sister, aged fourteen, developed phthisis, and died in the following January. During her illness the father developed a cough, and the mother suffered from pain in the abdomen and left ankle. Two daughters, aged twenty and ten, became ill, and the infant, aged two, became anæmic and rapidly emaciated. The mother was subsequently found to be suffering from tuberculous disease of the ankle, and the two girls and the baby from pulmonary phthisis. The baby died in January, having been ill three months. The father gradually got worse, and ultimately succumbed to phthisis. In March one of the boys, aged sixteen, was found to be suffering from pulmonary tuberculosis.

The cases just quoted place beyond all doubt not only the infectiousness, but the virulence which tubercle may occasionally assume. And yet, notwithstanding these and other cases, the infectiousness of tubercle is on the whole of a comparatively mild nature. Circumstances are, however, now and again in operation whereby the virulence of the tubercle bacillus becomes increased. We have two factors to deal with—an organism whose virulence can vary, also the individual whose resistance to the micro-organism may be increased or diminished in accordance with the influence of heredity, the immunity acquired by harbouring the bacillus in small numbers; also the opposite—viz. an increased susceptibility to the disease or anaphylaxia.

### The Place of Infection.

It is a question as to how far infection has played any notable part in spreading the disease in the wards of a large hospital. That flies are carriers of the disease, and therefore objectionable intermediaries, is now beyond all dispute. Last year I carried out with Dr. Slade, bacteriologist to the Royal Victoria Infirmary, a series of experiments to test the infectivity of the expired air and of the sputum of phthisical patients. I got three patients to speak into, read aloud for ten minutes into, and to cough into specially prepared agar-glycerine films from which cultures were made and emulsions injected into the bodies of guinea-pigs. In one case where the patient, a pronounced tuberculous subject, had coughed into the film thirty-seven times an emulsion injected into guinea-pigs did not give rise to tuberculosis; nor did it do so in the case of another guinea-pig, but in a third case the emulsion made from the droplets caught in the film during coughing was followed by miliary tuberculosis in which the liver and lungs were much implicated. Of equally infective power did I regard the expectoration of these three patients who coughed on to the prepared films, and yet only from one of them did tuberculosis in animals follow. Still even this is too high a percentage of risk to be run in a hospital by bringing susceptible persons within the range of infection from tuberculous patients. In order to test how far the blood of persons suffering from well-marked pulmonary phthisis might contain the tubercle

* *Control and Eradication of Tuberculosis*, by Many Authors, p. 12. (Edinburgh: Wm. Green and Sons.)

bacillus, Dr. Slade and I carried out several experiments, but not in one did a guinea-pig become tuberculous.

I must admit that in all these experiments I expected more serious results to have followed inoculation of animals. With ordinary care, therefore, as regards ventilation, cleanliness, removal and disinfection of the sputum, the risk of infection in the ward of an infirmary is not so great as to cause alarm; but the point rather is this : Is it wise to run any risk at all? What, too, is the best thing for the patient himself, and what is the best for others? There is also another side to the problem : Can the admission of tuberculous patients into a general hospital be altogether prevented; and, if so, is it desirable from a teaching point of view that they should be excluded? On the whole there is not the least doubt that the best results of the treatment of tuberculous patients are obtained by residence in sanatoria, and yet to sanatoria unqualified praise cannot be given, for results have not always come up to expectation. There is not the least doubt that those who adopt the open-air treatment of tuberculosis are proceeding on the most satisfactory and most hygienic lines. Compared with cattle which are housed during the winter months, and which thereby become susceptible to tubercle, the herds which live out-of-doors all the year round and roam about freely in the open do not suffer. An appeal is made to this fact in favour of the open-air life. But what of wood-pigeons sleeping on the tops of trees, breathing the purest air, and yet dying from tuberculosis? Such a circumstance weakens our belief in the efficacy of pure air. Notwithstanding all this, the crowded ward of a general hospital is not the ideal place for the treatment of tuberculosis. There is not the quantity nor the purity of the air the patient requires.

### Tuberculous Patients in General Hospitals.

With this mixed experience before us, we return to the question : Ought tuberculous patients to be admitted into a general hospital? And to that I must return the answer "No" and "Yes." You cannot, in fact, keep them out. Take, for example, acute miliary tuberculosis attended by high fever, and which is with difficulty differentiated from typhoid fever. Such a case may find its way into the ward of a general hospital, and I must admit that I have never seen any bad effects follow. Wherever housed, the patient is almost sure to succumb to his malady, and there is little likelihood of the disease spreading through the wards of an infirmary, since patients are not only admitted into the wards, but are treated and die without, in some instances, the diagnosis being accurately made until on the post-mortem table. I have never known of a case of acute miliary tuberculosis treated in a general hospital followed by the development of the disease in other patients in the wards. There are, too, cases of men and women who when at work or out walking are suddenly seized with hæmoptysis, so that should the blood-spitting be rather profuse and the shock great, it would be impossible from a humane point of view to refuse admission to such patients. Or, again, a patient is sent into hospital suffering from pleurisy with effusion. Seeing that such a large percentage of cases of pleurisy are tuberculous at the commencement, or become so afterwards, the admission of such a case into a general hospital cannot be refused. It is almost useless to discuss a problem like this, when post-mortem statistics show that in 70 per cent. or more of the bodies of persons dying in infirmaries from all causes, including accidents, there are evidences of cured tuberculosis. Take, again, peritoneal effusions. Who can say in the first instance what is the pathology of such cases? In young persons probably most of the effusions are tuberculous. They not only do well under treatment in hospital wards, but they do not spread the disease to others. Apart from bacillus coli, gonococcal and streptococcal infections of the uro-genital tracts in men and women, is not salpingitis with adherent tubes, also similar focal accumulations of pus in the kidney, frequently of tuberculous origin? When we deal with diseases of the alimentary canal, are we always sure of our diagnosis? Cases of tuberculous ulceration of the intestine and patients with enlarged glands in the abdomen find their way into hospitals, where their malady may be accurately diagnosed or not.

### The Dangers of Infection.

There are the two extremes of life at which tuberculosis may occur and the dangers of infection not be recognised : (1) Young children under two years of age with meningitis, and (2) old asthmatic and emphysematous patients over sixty years of age. Children under two years of age who are the subjects of pulmonary disease do not expectorate. Tuberculosis assumes many forms and underlies many anomalous affections not at first believed to be tuberculous, as seen in various types of joint disease primarily regarded as rheumatic, but which later experience has proved to be pulmonary osteoarthropathies. Tuberculosis is too subtle a malady, and its influences and ramifications are too widely spread to bring it under the unqualified ban of prohibited admission into a general infirmary, as the following illustration will show. A mason's labourer falls from a scaffolding and receives an injury to the wall of his chest. He is admitted into an infirmary suffering from fractured or injured ribs, pleurisy develops, and this is subsequently found to be tuberculous. The pleurisy may or may not have been tuberculous shortly after the injury, but it becomes so. Is such a patient to be refused admission into a general hospital?

It is another thing when we come to discuss the question of open forms of tuberculosis, cases in regard to which there is no difficulty of diagnosis, such as advanced pulmonary phthisis with abundant expectoration, also suppurating glands in the neck. I do not know whether there are instances, in the surgical wards, of patients, the subjects of suppurating glands, having conveyed the disease to others. It is not desirable that patients in advanced or rapidly advancing pulmonary phthisis should be admitted into the wards of a general hospital, but this is as much from the point of view that for the patient himself a crowded ward is not the best place for him nor for the other patients, whose night's rest may be disturbed by the frequent coughing of the intruder. The men or women in the ward must not only be protected from the phthisical patient, but the phthisical patient must himself be protected. The possibilities of infection cannot be ignored, for the opportunities are freely given to the inmates of the ward to sit beside and talk to a phthisical patient in the same ward, whose frequent cough and expectoration cannot but be without some risk to men and women whose own health at the time is not good. It may take months for a tuberculous infection to reveal itself, and as we cannot always follow the after-history of the patients who leave infirmaries, we cannot be quite sure whether all who may have been placed in the way of infection remain free from the disease. Years ago a larger number of cases of pulmonary phthisis was admitted into general hospitals than is admitted to-day. During my experience of thirty-four years as a hospital physician I can only recall two cases of possible tuberculous infection of one patient by another. I refer to cases of the disease developing under observation, for tubercle has no definite period of incubation, like most infectious diseases; hence the difficulty of estimating the actual amount of infection which may have taken place. We come, therefore, to this, that there is a large number of cases of tuberculosis which cannot be kept out of an infirmary, owing to imperfect diagnosis; there are many cases of concealed tuberculosis which are treated therein satisfactorily; also that special hospitals, annexes to general hospitals, and sanatoria are places wherein the best results from treatment are obtained. The exclusion of cases of tuberculous disease from teaching hospitals would mean a serious loss to the education of the medical student. Unless we are prepared to allow all our voluntary hospitals to be taken over by the State, I view with a certain amount of apprehension the desire of local Insurance Committees to subscribe for, and therefore to have control of, a certain number of beds in a general hospital; for as the needs of the public become greater the demands of the Committee will also increase, and thereby alter the character of the institution and the original intentions of those who founded the voluntary hospital. In the hospitals of Paris, which are all State-owned, Professor Landouzy reminds us that two thirds of the clientele are cases of tuberculosis.

### The Special Dispensary.

As regards other places for the treatment of the tuberculous, there is not the least doubt that a special dispensary, such as recommended by Sir Robert Philip, is the place where patients should first be sent to, and there undergo a process of sifting. This would save general hospitals being called upon to deal with at least a fairly large percentage of the cases, and would not only lighten the wards, but, by the nurses following up the cases to their homes, patients could be dealt with in special hospitals, if such exist, or in sanatoria where these have been provided.

If the prevention of tuberculosis is to be the main thing aimed at, the treatment of affected patients in a general hospital is not the best way to accomplish it. Tuberculosis has become a national problem; it is no longer a question of the individual or his family. It calls for treatment in the mass, and not in detail alone. It means not only medical care of the patient and his family, but consideration of the people as a whole. Special hospitals for the treatment of tuberculosis must therefore be established, provision being made for the hopeless cases as against those likely to benefit by treatment, and these hospitals will have to be maintained by the municipality or by the State. As the years roll on the State is becoming more and more the guardian of the health of the public. One means by which she can do good in this direction is by not neglecting infected children, for many cases of tuberculosis in the adult are the result of neglected infection dating back to childhood.

### The Effect of the Insurance Act.

There is considerable uncertainty and a degree of disquietude in the minds of many persons as to the influence the National Insurance Act is likely to have upon the future of our hospitals generally, and upon the treatment of tuberculous cases in the wards particularly. Local Insurance Committees with the wide powers assigned to them under the Act will prefer, it seems to me, to send all the hopeful cases they possibly can to sanatoria, while for hopeless cases special hospitals will have to be built. There will always be a certain number of tuberculous patients who will find their way into the wards of general hospitals or into the annexes. The Local Government Board is willing to pay any hospital for the admission and treatment of tuberculous patients, but while such financial assistance would be of the greatest help to institutions whose finances are low, the Local Government Board would insist upon the right to enter these hospitals at any time, to control supervision, and possibly, also, to direct treatment. Besides, as already stated in an earlier part of this address, the Board might flood the hospital with tuberculous cases and thereby alter the character of the institution. In large industrial centres where infirmaries are well supported by the working-men it is more than likely, seeing that working-men are paying to panel doctors, that they may refuse to contribute to the infirmary, but this attitude is hardly likely to be long maintained since for severe and protracted illness, also for surgical diseases, the panel doctor has neither the time, the means, nor the surgical training and experience to enable him to cope with such maladies. Nor would local Insurance Committees encourage the men to take up this position. The working-classes will only get from the panel doctors what they pay for and what the Act allows. As it is clearly to the advantage of the working-classes to continue their contributions to general hospitals, the National Insurance Act is scarcely likely to affect the future of infirmaries in our large towns, while so far as tuberculous patients are concerned the Act will tend to remove these patients from general hospitals and to place them in sanatoria. We do not want nor has the time yet come for our voluntary hospitals to be managed by the State.

